# Pyruvate kinase is necessary for *Brucella abortus* full virulence in BALB/c mouse

**DOI:** 10.1186/s13567-016-0372-7

**Published:** 2016-08-25

**Authors:** Jianpeng Gao, Mingxing Tian, Yanqing Bao, Peng Li, Jiameng Liu, Chan Ding, Shaohui Wang, Tao Li, Shengqing Yu

**Affiliations:** Shanghai Veterinary Research Institute, Chinese Academy of Agricultural Sciences (CAAS), Shanghai, People’s Republic of China

## Abstract

Brucellosis, caused by a facultative intracellular pathogen *Brucella*, is one of the most prevalent zoonosis worldwide. Host infection relies on several uncanonical virulence factors. A recent research hotpot is the links between carbon metabolism and bacterial virulence. In this study, we found that a carbon metabolism-related pyruvate kinase (Pyk) encoded by *pyk* gene (locus tag BAB_RS24320) was associated with *Brucella* virulence. Determination of bacterial growth curves and resistance to environmental stress factors showed that Pyk plays an important role in *B. abortus* growth, especially under the conditions of nutrition deprivation, and resistance to oxidative stress. Additionally, cell infection assay showed that Pyk is necessary for *B. abortus* survival and evading fusion with lysosomes within RAW264.7 cells. Moreover, animal experiments exhibited that the Pyk deletion significantly reduced *B. abortus* virulence in a mouse infection model. Our results elucidated the role of the Pyk in *B. abortu*s virulence and provided information for further investigation of *Brucella* virulence associated carbon metabolism.

## Introduction

Brucellosis, which is characterized by a febrile disease and infectious abortion in animals, is caused by *Brucella spp*, and remains one of the most common zoonotic diseases worldwide [1]. As the threat of *Brucella* to human health and animal husbandry has continued to increase in recent years, this disease has attracted ever more attention from scientific community and governments in affected areas throughout the world [2]. Moreover, *Brucella* strains have the potential to be used in biological warfare [3]. Therefore, in-depth research of *Brucella* virulence is truly pressing and meaningful.

*Brucella*, a genus of gram-negative bacteria, can grow in vitro and multiply in both phagocytic cells and non-phagocytic cells. Besides, it has evolved an amazing ability to evade host immunity and establish chronic infection. However, *Brucella* has no classic virulence factors, such as exotoxins, cytolysins, capsules, fimbria, and endotoxic lipopolysaccharide (LPS) [4]. Up to date, several virulence-associated factors have been identified, which are indispensable for the survival of *Brucella* in host cells, including a type IV secretion system, a two-component regulatory system composed of regulatory (BvrR) and sensory (BvrS) proteins, cyclic β-1,2-glucans, superoxide dismutase, catalase and urease [4]. Recently, much progress has been made in the study of possible links between carbon metabolism and intracellular bacterial virulence, especially in model intracellular pathogens, such as *Listeria monocytogenes*, *Shigella flexneri*, *Salmonella entericaserovar typhimurium* and *Mycobacterium tuberculosis* [5–8].

After entering the host cells, intracellular pathogens have to adjust their metabolism to the environmental conditions encountered in its intracellular replicative niche, including low oxygen and nutrient levels, acidic pH and so on [9]. In this process, the regulation of carbon metabolism may directly or indirectly influence the expression of the virulence genes within the host cell and, hence, pathogen virulence [5]. In *Brucella*, many genes reportedly associated with carbon metabolism are necessary for virulence, such as 6-phosphogluconate dehydrogenase (*gnd*), phosphoglucose isomerase (*pgi*), pyruvate carboxylase (*pyc*) and some erythritol catabolism genes (e.g., *eryB*, *eryC*) [9–11]. According to the previous reports, it has been accepted that glucose is mainly catabolized in *Brucella* through the pentose phosphate pathway in conjunction with the tricarboxylic acid (TCA) cycle [12, 13]. Of these, pyruvate is one of the more important substances that connects the glucose catabolism pathway with TCA cycle, in which several related genes have been shown to be necessary for *Brucella* virulence. Pyruvate phosphate dikinase (Ppdk), which is involved in classical gluconeogenesis, is required for full virulence in *B. abortus* [14]. Pyc is an enzyme of the ligase class that catalyzes the irreversible carboxylation of pyruvate to oxaloacetate, which was identified as a virulence-related gene by random mutagenesis [9]. It has been suggested that pyruvate catabolism plays an essential role in the full virulence of *Brucella*.

Our recent study found that pyruvate kinase, which is encoded by the *pyk* gene (gene locus BAB_RS24320) is associated with *B. abortus* virulence by PCR-based on signature-tagged mutagenesis (data unpublished). Pyk catalyzes the synthesis of pyruvate from phosphoenolpyruvate (PEP), that is, adenosine diphosphate + phosphoenolpyruvate = adenosine triphosphate + pyruvate, which is required for glucose catabolism through the glycolysis pathway. In this study, we further investigated the role of Pyk on *B abortus* virulence and found that Pyk plays important roles on the bacterial resistance to oxidative stress, escaping from fusion with lysosome within macrophages, and establishing infection in BALB/c mouse.

## Materials and methods

### Ethic statement

This study was performed in strict accordance with the recommendations in the Guide for the Care and Use of Laboratory Animals of the Institutional Animal Care and Use Committee guidelines set by Shanghai Veterinary Research Institute, the Chinese Academy of Agricultural Sciences (CAAS). Mice (SLAC Experimental Animal Inc., Shanghai, China) were housed in cages with water and food ad libitum under biosafety conditions. Animal handling and procedures were approved by the Committee on the Ethics of Animal Experiments of Shanghai Veterinary Research Institute, CAAS (permit number: SHVRI-mo-0175).

### Bacterial strains and growth conditions

*B. abortus* strain S2308 was obtained from the Chinese Veterinary Culture Collection Center (Beijing, China) and routinely grown in tryptic soy broth (TSB) (Difco™, BD BioSciences, Franklin Lakes, NJ, USA) or tryptic soy agar (TSA). *Escherichia coli* strain DH5α was grown on Luria–Bertani medium. When appropriate, 100 μg/mL of ampicillin or 20 μg/mL of chloramphenicol (Sigma–Aldrich Corporation, St. Louis, MO, USA) respectively, were added. All strains and plasmids used in the study are listed in Table 1.

**Table 1:** **Strains and plasmids used in the study**

Strains and plasmids	Characteristics	Source
*Brucella abortus*		
S2308	Wild type strain; Smooth phenotype	ATCC
∆*pyk*	*pyk* gene deletion mutant strain; Smooth phenotype	This study
∆*pyk*(Pyk-3 × Flag)	Cm^r^; complementation strain; ∆*pyk* carrying the complementation plasmid pBBR-*pyk*-3 × Flag; Smooth phenotype	This study
*Escherichia coli*		
DH5α	F^−^ φ80*lac*Z∆M15 ∆(*lac*ZYA-*arg*F)U169 *rec*A1 *end*A1 *hsd*R17(r_k_^−^, m_k_^+^) *pho*A *sup*E44 *thi*-1 *gyr*A96 *rel*A1 λ^−^	Invitrogen
*Plasmids*		
pBBR1MCS1	Cm^r^; Broad-host-range cloning vector	[17]
pSC	Amp^r^; pUC19 plasmid containing *SacB* gene	[16]
pSC-∆*pyk*	Amp^r^; pSC plasmid containing the ∆*pyk* fragment; used to construct deletion strain	This study
p3 × Flag-CMV-14	Amp^r^; eukaryotic expression plasmid	Sigma-Aldrich
pBBR-*pyk*-3 × Flag	Cm^r^; pBBR1MCS1 containing the *pyk* gene flanked by its upstream and downstream regions containing a C-terminal 3 × flag tag.	This study

### Construction of suicide and complementation plasmids

Suicide plasmids were constructed using an overlap PCR assay, as previously reported [15]. Briefly, efficient primers were designed for amplification of a 947-bp upstream fragment and a 995-bp downstream fragment of the *pyk* gene by a first round of PCR. After purification by gel extraction, the recovered products containing joined flanking sequences were used as templates for a second round of overlap PCR. Then, the PCR product was gel purified, digested with *Xba*I, and cloned into the *Xba*I-digested pSC plasmid [15, 16]. The recombinant suicide plasmid pSC-Δ*pyk* was transformed into competent DH5α cells (Invitrogen Corporation, Carlsbad, CA, USA) for propagation and then extracted to construct the mutants.

In order to construct the complementation plasmid, the *pyk* gene was amplified by PCR using the primer pair Cpyk-F/Cpyk-R, the product was recovered, digested with the restriction enzymes *Kpn*I and *Bam*HI, and inserted into the p3 × Flag-CMV-14 plasmid (Sigma-Aldrich) to construct the recombinant plasmid p3 × Flag-*pyk*, and then the inserted *pyk* fragment with a 3 × Flag tag fragment was amplified by PCR using the primers Cpyk-F and 3 × Flag-R from the p3 × Flag-*pyk* plasmid, the fragment was digested with *Kpn*I and *Xba*I, and inserted into the pBBR1-MCS1 plasmid [17]. The complementation plasmid was designated as pBBR-*pyk*-3 × Flag.

### Construction of the *pyk* mutant and the complementation strain

The *pyk* mutant was constructed by allelic replacement using a two-step strategy, as previously reported [18]. Briefly, competent *B. abortus* strain S2308 cells were prepared through two washes with ice-cold sterile water and suicide plasmid (0.5–1.0 μg) was transformed into competent cells by electroporation. The single exchanged recombinants were selected by plating on TSA containing ampicillin, and then colonies were inoculated into TSB without antibiotics. The second exchanged recombinants were selected by plating on TSA containing 5% sucrose. All colonies were selected and verified by PCR amplification.

The complementation strain was constructed by transforming the plasmid pBBR-*pyk*-3 × Flag by electroporation, as described above, and the recombinant strain was designated as Δ*pyk*(Pyk-3 × Flag).

### Western blotting analysis

Western blotting analysis was performed to verify the complementation strain Δ*pyk*(Pyk-3 × Flag). The fresh cultural Δ*pyk*(Pyk-3 × Flag) and S2308 strains were harvested, respectively, by centrifugation 8000 × *g* for 5 min, the pellets were suspended in a mixture of deionized water and 2 × SDS-PAGE loading buffer (Beyotime Institute of Biotechnology, Shanghai, China), and boiled for 5 min. Proteins were separated on a 12.5% SDS polyacrylamide gel and transferred onto nitrocellulose membrane (Whatman^®^; Sigma-Aldrich). The membrane was blocked for 2 h at room temperature with phosphate-buffered saline (PBS; hyclone) contained 5% skim milk, and then incubated with mouse anti-Flag monoclonal antibody (Sigma-Aldrich) in PBST (PBS containing 0.2% tween-20) overnight at 4 °C. After washing five times with PBST, the membrane was incubated with IRDye 680RD-conjugated donkey anti-mouse polyclonal antibody (LI-COR Biosciences, Lincoln, NE, USA) in PBST for 1 h at room temperature. After washing five times with PBST, protein bands were detected using the Odyssey Infrared Imaging System (LI-COR Biosciences).

### RNA isolation and real-time qRT-PCR

To verify the transcriptional level of the *pyk* upstream gene *bab_RS24315* and the downstream gene *bab_RS24325*, total RNA was extracted from both S2308 and the *pyk* mutant by TRlzol reagent (invitrogen), and the bacterial genomic contamination was removed by the TURBO DNA-free kit (Ambion^®^; Invitrogen). The RNA was quantified by absorption at 260 nm using a NanoVue™ plus spectrophotometer (GE Healthcare Life Sciences, Logan, UT, USA). Then, qRT-PCR was carried out using GoTaq qPCR Master Mix (Promega, Fitchburg, WI, USA) and the Mastercycler ep realplex Real-Time PCR system (Eppendorf, Hamburg, Germany). For each gene, PCRs were performed in triplicate and relative transcription levels were determined by the 2^−∆∆Ct^ method using glyceraldehyde phosphate dehydrogenase (*gapdh*) as an internal control for data normalization. All primers used for real-time qRT-PCR are listed in Table 2.

**Table 2:** **Primers used in the study**

Primers	Oligonucleotide sequence (5′ to 3′)	Target genes	Products (bp)
Cpyk-F	GGGGTACCTTGTCCAATATAAAGCGATGAC *Kpn*I, underlined	The *pyk* containing the promoter region	1934
Cpyk-R	CGGGATCCAATGCCGGATTTTCCGTCAGCG *Bam*HI, underlined		
3 × Flag-R	GCTCTAGACAGGGATGCCACCCGGGATC *Xba*I, underlined	The 3 × Flag tag of p3 × Flag-CMV-14	
pyk-UF	CGGGATCCCGGGGGTTATGGAAAGCAACT	The upstream fragment of *pyk*	947
pyk-UR	TGACGACGCAATGCAGGCTCGAGGGTGAAGGTCTGG		
pyk -DF	CCAGACCTTCACCCTCGAGCCTGCATTGCGTCGTCA	The downstream fragment of *pyk*	995
pyk- DR	CGGGATCCCGTACGGGTGCGGGTGTTTC		
In-pyk-F	ATGCCGTGCTGAAGGAAGAG	The inside fragment of *pyk* gene	493
In-pyk-R	GCGTCAATGATGGTCGAATAGG		
Out-pyk-F	GGGGTACCCCGACGGTGGGAAGGCAAAG	The outside fragment of *pyk* gene	1885
Out-pyk-R	CGGGATCCCGGAGCGGCTCCAGAAATCG		
RT-pyk-F	AAAACTGCATCTGGTGGCTG	*pyk*	207
RT-pyk-R	GGGCGCTGGATAAAGGAAAG		
RT-upstream-F	ATGACATCAATCGCACGCTG	*bab_RS24315*	161
RT-upstream-R	GAAATTCTTTTGGGCGTCGC		
RT-downstream-F	AAGCTGCAAAACCCTGATCG	*bab_RS24325*	209
RT-downstream-R	AGCTTGATTGTTCCCCGGTA		
RT-GAPDH-F	GACATTCAGGTCGTCGCCATCA	*gapdh*	188
RT-GAPDH-R	TCTTCCTTCCACGGCAGTTCGG		

### Growth curve in TSB and minimal medium

Bacterial growth was measured at an optical density at 600 nm (OD_600_). For growth curve analysis, bacterial strains were incubated in TSB for 24 h, and then diluted with TSB to an OD_600_ value of 0.01 and cultured in a rotary shaker (200 rpm) at 37 °C. Cultures were taken at appropriate interval and OD_600_ values were recorded.

Bacterial growth in minimal medium was measured as described above except minimal medium was used as the diluting solution. Components of the minimal medium were glucose (10 g/L), yeast extract (1 g/L), (NH_4_)_2_SO_4_ (13.2 g/L), Na_2_S_2_O_3_·5H_2_O (0.1 g/L), MgSO_4_ (10 mg/L), MnSO_4_ (0.1 mg/L), NaCl (5 g/L) and KH_2_PO_4_ (3 g/L). The pH was adjusted to 6.8 to 7 [19]. In order to further evaluate the effect of metabolic product (sodium pyruvate) by Pyk catalysis, sodium pyruvate was added into the minimal medium with concentrations of 1, 5 or 10 mM or glucose was replaced by pyruvate as sole carbon source at final concentration of 200 mM in minimal medium.

### Cell infection assay

RAW 264.7 macrophages were used to assess the ability of the *pyk* mutant to survive intracellularly. The experiment was performed as previously reported [20]. Briefly, the cells were seed in 24-well plates and grown in Dulbecco’s Modified Eagle Medium (DMEM) (Hyclone™; GE Healthcare) supplemented with 10% fetal bovine serum (FBS) (Gibco^®^; Invitrogen) at 37 °C with 5% CO_2_ for 24 h. The cell monolayer was washed twice with DMEM and infected with *B. abortus* S2308 or the *pyk* mutant at a multiplicity of infection of 100. Bacteria were centrifuged onto the cells at 400 × *g* for 10 min and the cells were then incubated at 37 °C for 1 h. Non-adherent bacteria were removed by rinsing the wells twice with PBS. To kill extra-cellular bacteria, the cells were incubated with DMEM containing gentamicin (100 μg/mL) for an additional 1 h, washed twice with PBS and the medium was replaced with DMEM containing 2% FBS and 20 μg/mL gentamicin. At 2, 8, 24 and 48 h post-infection, the macrophages were lysed with 0.2% Triton X-100 in sterile water and the live bacteria were enumerated by plating on TSA plate. All assays were performed in triplicate and repeated at least three times and the results are the averages from at triplicate infection samples.

### Immunofluorescence assay

RAW264.7 cells cultured on glass coverslips (Thermo Fisher Scientific, Waltham, MA, USA) were infected with *Brucella* at a multiplicity of infection of 100. The infected cells were fixed with 3.7% (w/v) paraformaldehyde at 4 °C for 24 h post-infection. The immunofluorescence assay was performed as previously described [15] using rabbit anti-*Brucella* serum (diluted 1:1000) and Rat anti-LAMP-1 monoclonal antibody [1D4B] (diluted 1:500, Abcam, Cambridge, MA, USA) as the primary antibody, and Alexa Fluor 488-conjugated goat anti-rabbit IgG (diluted 1:1000) and Alexa Fluor 555-conjugated goat anti-rat lgG (diluted 1:1000) (Invitrogen) as the secondary antibody. The coverslips were mounted onto glass slides using Eukitt quick-hardening mounting medium for microscopy (Sigma-Aldrich) and the cells were observed under a Nikon Eclipse 80i microscope (Nikon Corporation, Tokyo, Japan) with 100× oil immersion objective. Images were saved in TIFF format and imported to Adobe Photoshop CS4 (Adobe Systems Incorporated, San Jose, CA, USA), where they were merged using RGB format. To determine the percentage of bacteria positive for the lysosome marker LAMP-1, 100 intracellular bacteria were counted randomly. The assays were performed in triplicate.

### Stress resistance assay

The S2308 strain and the *pyk* mutant were cultured to mid-logarithmic phase (the value of OD_600_ ≈ 1.0) at 37 °C in TSB medium, and then the bacterial suspension was diluted with PBS and adjusted to a concentration to 4 × 10^5^ colony-forming units (CFU)/mL. Afterward, 50 μL of bacterial suspension was mixed with 50 μL of the appropriate reagent. The effects of various stress factors were tested as follows. H_2_O_2_ was used to determine sensitivity to oxidative stress and added at final concentrations of 0.5, 1 or 1.5 mM. Polymyxin B at concentrations of 50, 100 or 200 μg/mL was used to test sensitivity to cationic bactericidal peptides. Bovine serum and heat-inactivated bovine serum were used to assess resistance to natural serum killing with a bactericidal activity assay. In all tested groups, a negative-control group was introduced by adding 50 μL of PBS to the same bacterial suspension. After exposure for 1 h at 37 °C, the mixtures were rapidly diluted and plated on TSA plates to determine viability. Results are expressed as the mean percentage of the negative control from independent triplicate samples.

To determine the ability of the *pyk* mutant to resist acidic pH, an acid tolerance assay was performed as previously described with some modifications [20]. Briefly, the bacterial suspension of the S2308 strain and the *pyk* mutant were cultured and diluted to 2 × 10^7^ CFU/mL in TSB with pH of 7.3, 5.5 or 4.5. After 1 h of incubation at 37 °C, cells were serially diluted and plated on TSA to determine the number of bacterial CFU. The percentage of surviving bacteria was calculated with respect to CFU obtained from bacteria incubated in TSB at pH 7.3 (100% survival).

### Infection of mice

Virulence assay using BALB/c mice was performed as reported previously with some modifications [16]. Briefly, 6-week-old female BALB/c mice (*n* = 6 per group) were intraperitoneally inoculated with 0.1 mL of suspension containing 1 × 10^6^ CFU of the S2308 strain, the *pyk* mutant or the complementation strain. The survival of the bacteria in mice was evaluated by bacterial enumeration in the spleens at different time points post-infection. At 1 or 5 weeks post-infection, mice were sacrificed by cervical dislocation. The spleens were harvested, and homogenized in 5 mL of PBS-0.2% Triton X-100. After that, serial dilutions of the homogenates were made and plated on TSA plates to determine the bacterial loads. The data are expressed as the log_10_ CFU per spleen. Besides, spleen weight was measured to evaluate splenomegaly.

### Statistical analysis

Statistical analysis was performed using GraphPad Prism software 5.0 (GraphPad Software Inc., La Jolla, CA, USA). Statistical significance was determined by either an unpaired or two-tailed Student’s *t* test, or in the case of groups, a one-way analysis of variance followed by the Tukey’s test. A probability (*p*) values of ≤0.05 were considered significant.

## Results

### The *pyk* mutant and the complementation strain were successfully constructed

**Figure 1:**
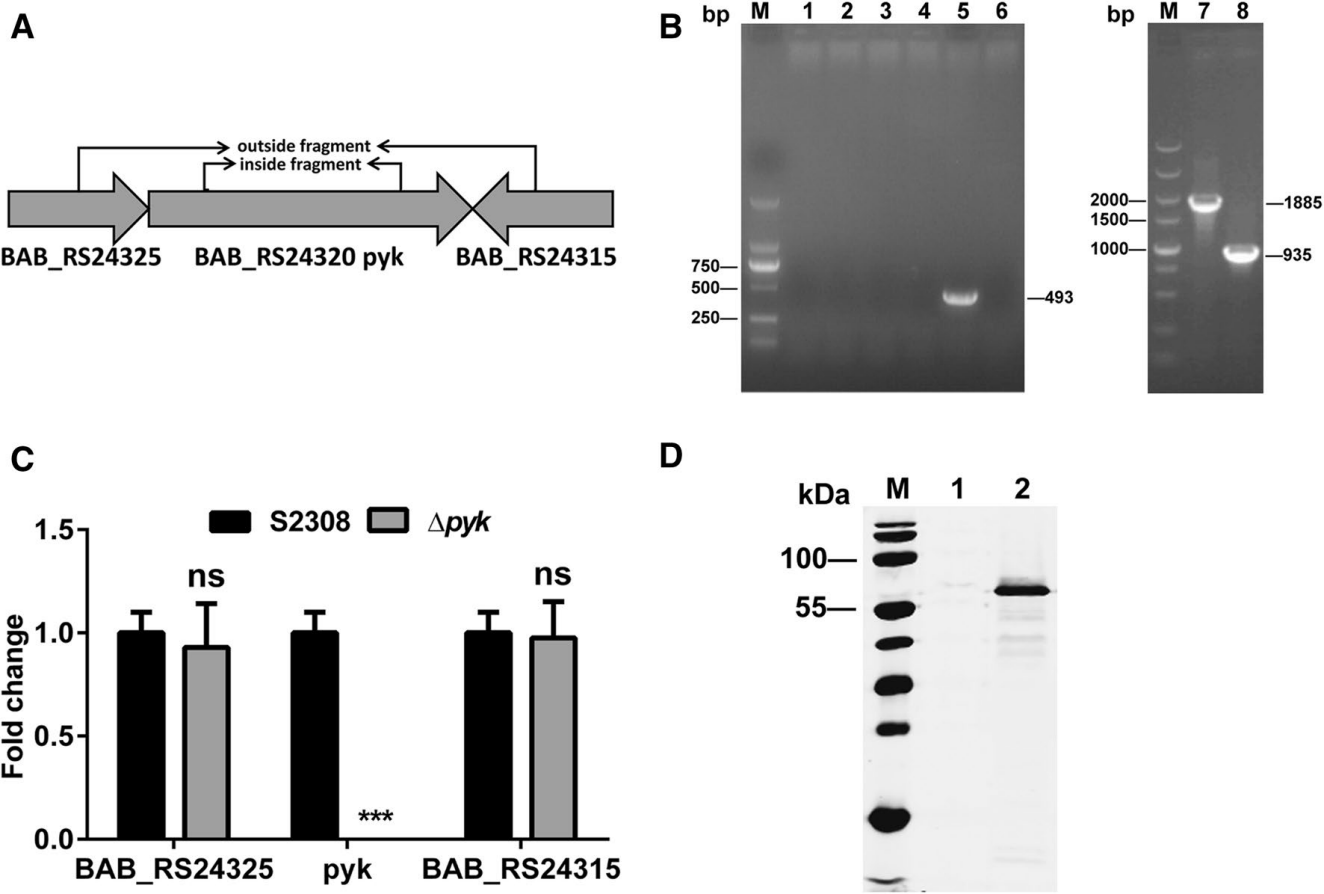
**Identification of the** ***pyk*** **mutant and the complementation strain. A** A schematic of the *pyk* gene and its flanking genes. The inside and outside fragments amplified using respective primers are indicated. **B** PCR amplification confirmed deletion of the *pyk* gene in the *pyk* mutant. Lane M: DNA marker DL2000 (Takara Bio, Inc., Shiga, Japan). Lanes 1–6: amplification of the inside fragment. Lanes 1–4: four different clones of the *pyk* mutant, no fragment was amplified. Lane 5: the S2308 strain (positive control), a 493-bp fragment was amplified; Lane 6: sterile water (negative control). Lanes 7 and 8: amplification of the outside fragment. Lane 7: the S2308 strain (positive control), a 1885-bp fragment was amplified; Lane 8: the *pyk* mutant, a 935-bp fragment was amplified. **C** Real-time qRT-PCR confirmed the deletion of the *pyk* gene at the transcriptional level. The *pyk* deletion did not affect expression of the flanking genes. The gene transcriptional level of the *pyk* mutant was compared with that of the S2308 strain. ***p ≤ 0.001, ns: no significant difference. **D** Western blotting confirmed expression of *pyk* in the complementation strain Δ*pyk*(Pyk-3 × Flag). Lane M: prestained protein ladder (Thermo Fisher Scientific); Lane 1: the S2308 strain (negative control), no flag expression was detected; Lane 2: the complementation strain Δ*pyk*(Pyk-3 × Flag), about 55 kDa of the flag expression product was shown.

The *pyk* mutant was verified by PCR amplification of the inside and outside fragments using respective primers (Figure 1A). The results indicated that a 493-bp inside fragment and a 1885-bp outside fragment were amplified from the S2308 strain, respectively. However, the inside fragment was not amplified from the *pyk* mutant and the size of outside fragment was obviously shorter than that of S2308 due to deletion of the *pyk* gene (Figure 1B). In order to further identify the mutant and exclude the possible polar effects of *pyk* deletion, qRT-PCR was performed to quantify the expression of *pyk* and its flanking genes at the transcriptional level. The results showed that the *pyk* gene was not transcribed in the *pyk* mutant, and transcription of the upstream and downstream genes was not affected (Figure 1C). In addition, the complementation strain Δ*pyk*(Pyk-3 × Flag) was verified by western blotting, as shown in Figure 1D, Pyk-3 × Flag was successfully expressed in the *pyk* mutant.

### Pyk is required for intracellular replication and trafficking of *B. abortus*

**Figure 2:**
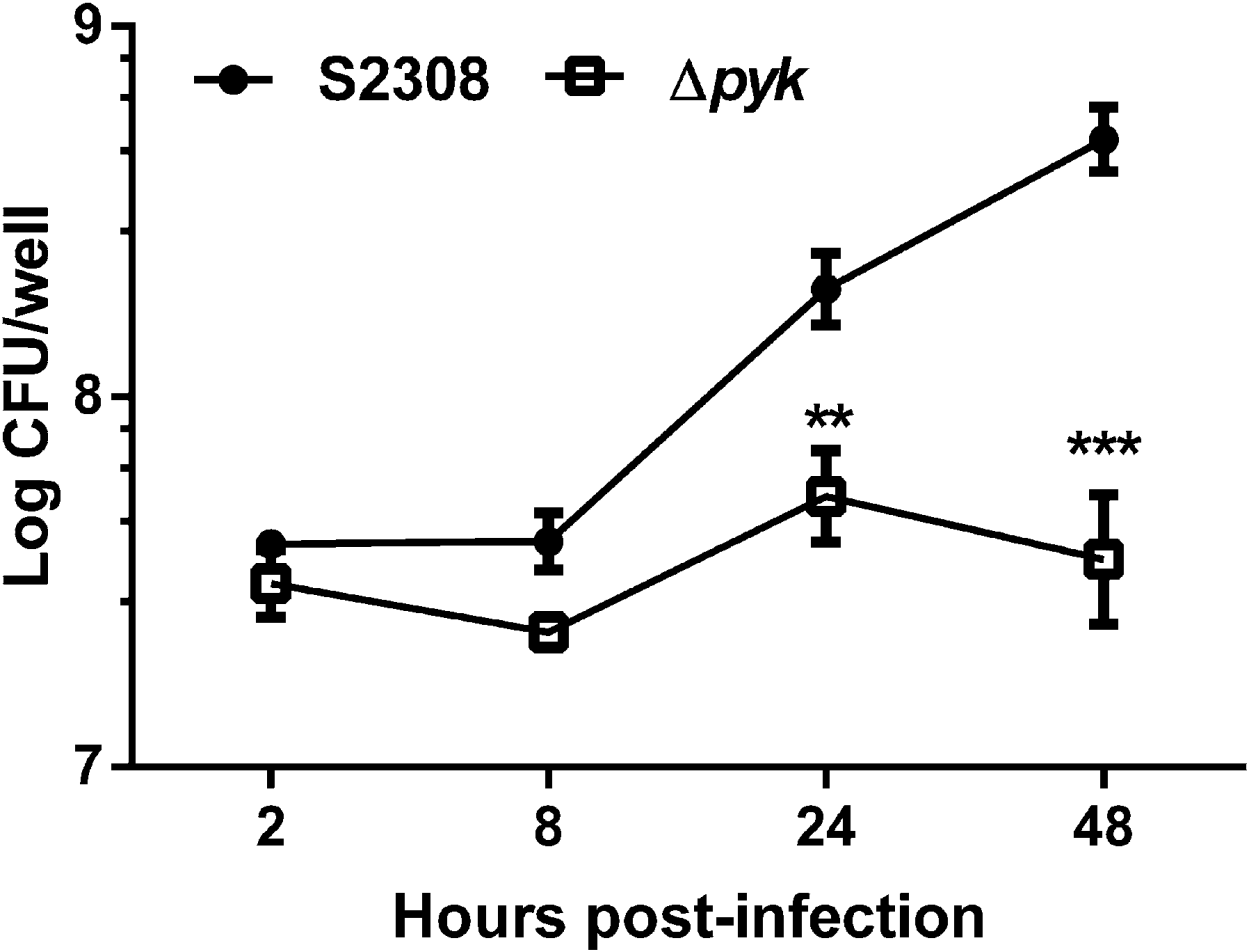
**Intracellular survival within RAW 264.7 macrophages of the S2308 strain and the** ***pyk*** **mutant.** Values are means ± standard errors for triplicate infection samples, and the experiments were repeated three times with similar results. **p ≤ 0.01, ***p ≤ 0.001.

**Figure 3:**
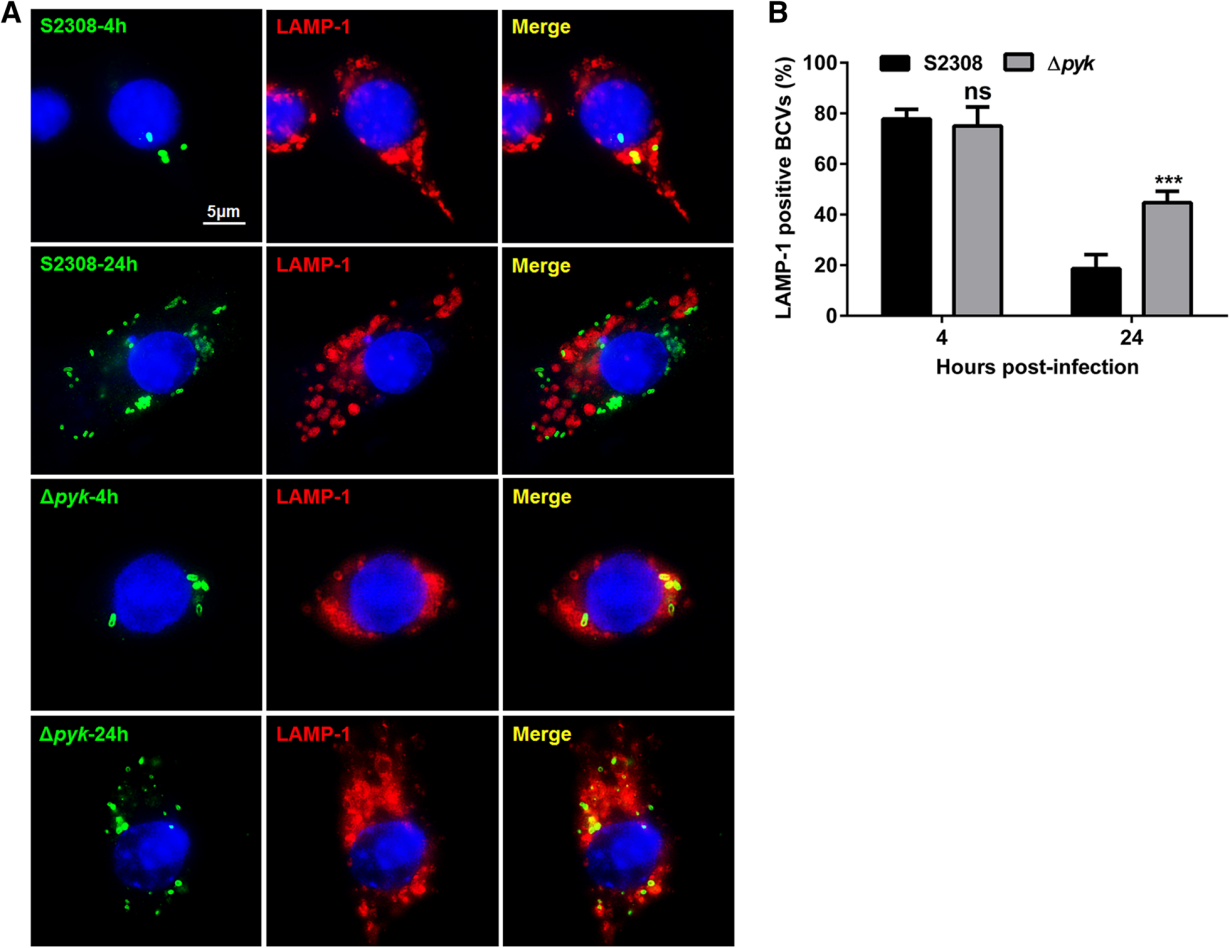
**The** ***pyk*** **mutant was not able to efficiently exclude lysosome-associated membrane protein-1 within RAW264.7 cells. A** Representative images of LAMP-1-positive and -negative BCVs. **B** Determination of the LAMP-1 positive BCVs at 4 and 24 h post-infection of the S2308 strain and the *pyk* mutant. The percentage of positive BCVs per 100 random BCVs is shown. ***p ≤ 0.001. ns: no significant difference.

To determine the effect of the *pyk* gene in intracellular survival, the ability of the *pyk* mutant to survive within macrophages was assessed at 2, 8, 24 and 48 h post-infection. As shown in Figure 2 there was no significant difference in intracellular survival between the S2308 strain and the *pyk* mutant at 2 and 8 h post-infection, indicating a similar ability of both strains to invade macrophages. However, a marked decrease in bacterial recovery from RAW264.7 cells infected with the pyk mutant, as compared with that of S2308 infected cells at 24 and 48 h post-infection (Figure 2). These results encouraged us to determine if the difference observed in the intracellular viable counts was a consequence of increased degradation or inactivation of the mutant. To this end, we determined the number of LAMP-1-positive *Brucella*-containing vacuoles (BCVs) at 4 and 24 h post-infection of RAW264.7 cells with the *pyk* mutant and the S2308 strain. As shown in Figure 3, the mutant showed a reduced capacity to exclude the lysosome marker LAMP-1 at 24 h post-infection (about 45% co-localization), as compared with the S2308 strain (about 20% co-localization), indicating that deletion of *pyk* decreased the ability of the *Brucella* to avoid the fusion of the BCV with lysosome.

### Deletion of *pyk* in *Brucella* impaired its growth in TSB and in minimal medium

**Figure 4:**
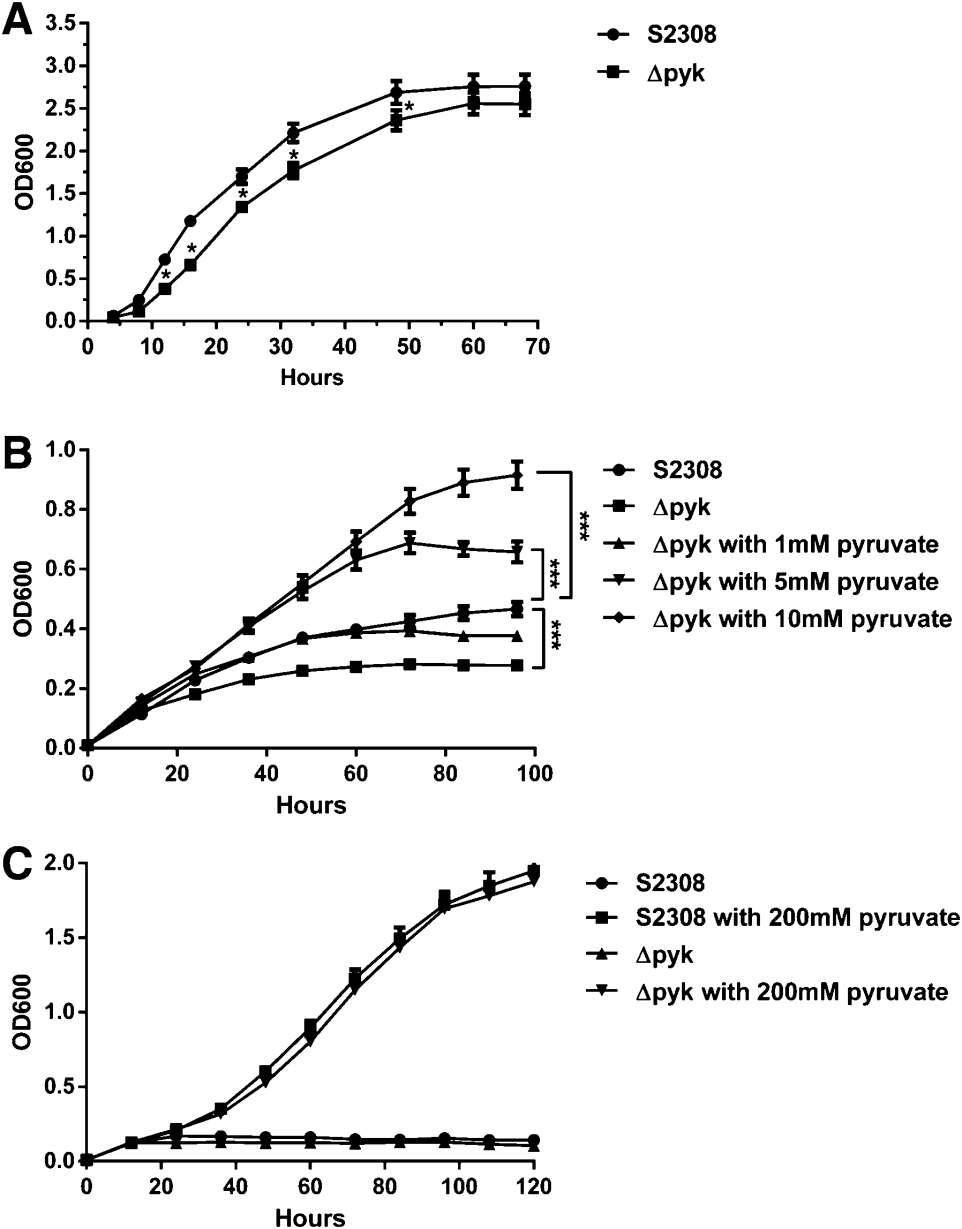
**Bacterial growth was affected by** ***pyk*** **deletion and recovered by supplementation with sodium pyruvate. A** Growth curves in TSB. **B** Growth curves in minimal medium with or without sodium pyruvate. **C** Growth curves in minimal medium without glucose or replaced glucose by pyruvate as sole carbon source at concentration of 200 mM. Each point represents the mean for triplicate samples (error bars are within the size of the symbols). The experiment was repeated three times with similar results. *p ≤ 0.05; ***p ≤ 0.001.

The Pyk is an important protein in glycolysis, and catalyzation of PEP to pyruvate, accompanied by the release of ATP. To further confirm the role of pyruvate in *Brucella* growth, we compared the bacterial growth of the *pyk* mutant with that of the S2308 strain in rich TSB and in minimal medium. As shown in Figure 4A, growth of the *pyk* mutant was slightly reduced at the logarithmic phase, but reached a similar stationary phase in rich TSB in comparison with the S2308 strain. However, in minimal medium the *pyk* mutant exhibited a significant growth defect throughout the growth stage, as compared with the S2308 strain (Figure 4B), suggesting that Pyk plays an important role in *Brucella* growth, especially under the condition of nutrition deprivation. To further assess the effect of pyruvate synthesis on *Brucella* growth, we assessed the ability of the *Brucella* to growth in minimal medium containing different concentrations of pyruvate. As shown in Figure 4B, growth of the *pyk* mutant restored to a similar level of the S2308 strain supplemented with pyruvate at final concentration of 1 mM, when supplemented with 5 or 10 mM of pyruvate, the growth of the mutant was faster than that of the S2308 strain. To further confirm the role of pyruvate in *Brucella* growth, we replaced glucose by pyruvate as sole carbon source in minimal medium at the concentration of 200 mM, no significant difference was found between the growth of the mutant and the S2308 strain (Figure 3C). These results revealed that the Pyk is necessary for *Brucella* growth, especially under the condition of nutrition deprivation, and the growth defect was due to the loss of pyruvate synthesis catalyzed by Pyk.

### The *pyk* mutant exhibited greater sensitivity to H_2_O_2_

**Figure 5:**
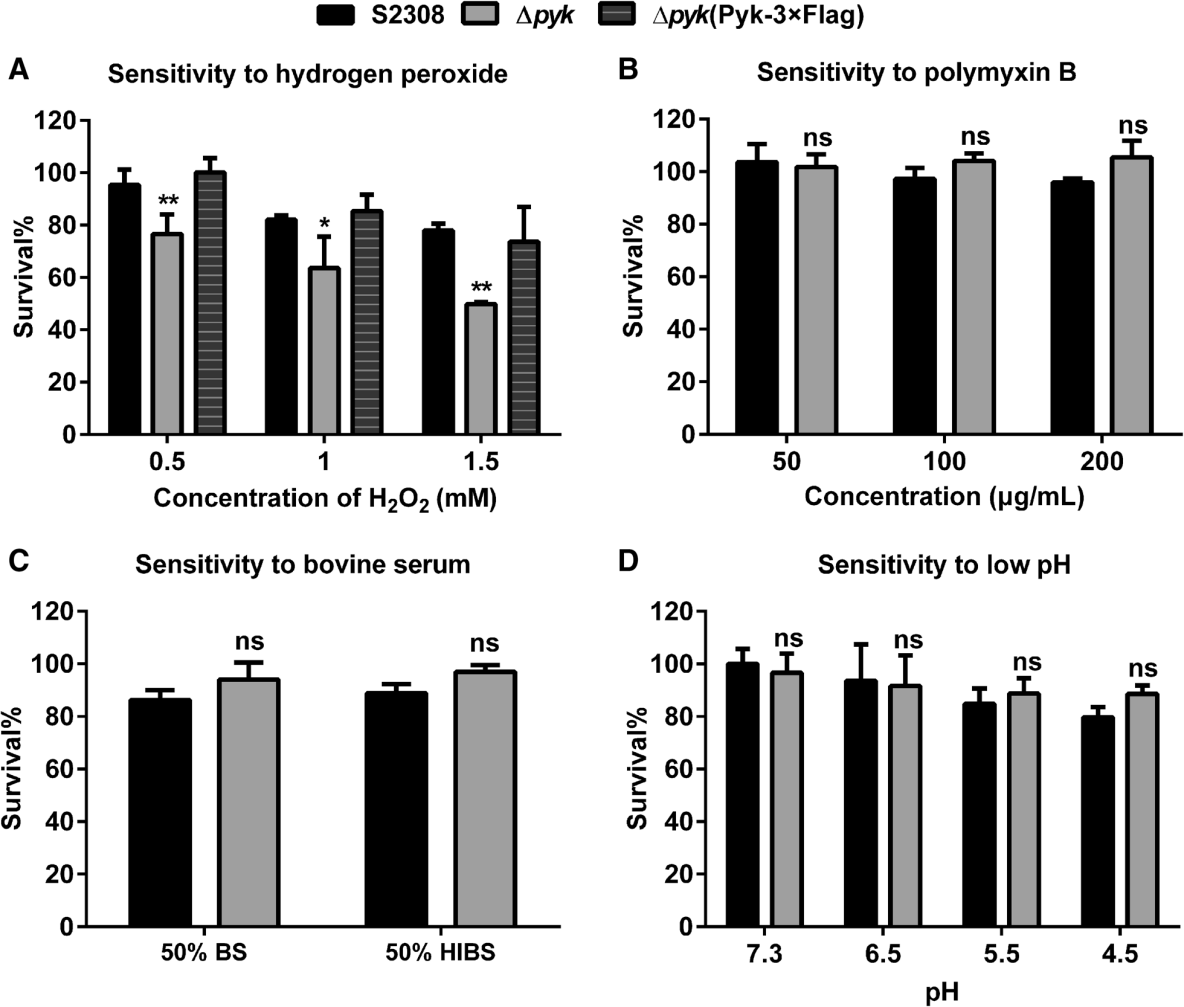
**Stress resistance assays. A** Sensitivity to H_2_O_2_ at concentrations of 0.5, 1 or 1.5 mM. **B** Sensitivity to polymyxin B at concentrations of 50, 100 or 200 μg/mL. **C** Sensitivity to bovine serum (BS) and heat inactivated bovine serum (HIBS). **D** Resistance to low pH. Values are means ± standard errors for triplicate samples. * *p* ≤ 0.05; **p ≤ 0.01; ***p ≤ 0.001; ns: no significant difference.

For *Brucella spp.* to successfully infect and establish chronic infection in their preferred host, the bacteria must have the ability to resist host bactericidal activity from the innate immune response, such as reactive oxygen and nitrogen species, bactericidal peptides, and low PH within macrophages. To this end, we assessed the ability of the *pyk* mutant to resist environmental stress factors. The results showed that the *pyk* mutant displayed higher sensitivity to different concentrations of H_2_O_2_, as compared with the S2308 strain. At 1.5 mM H_2_O_2_ exposure for 1 h, the mutant exhibited about 50% survival in comparison with 80% survival of the S2308 strain, and the mutant harboring the complementation plasmid pBBR-*pyk*-3 × Flag recovered resistance to H_2_O_2_ (Figure 5A). However, the mutant and the S2308 strain showed similar sensitivity to polymyxin B, bovine serum and low PH (Figures 5B–D).

### The *pyk* mutant was attenuated in mice

**Figure 6:**
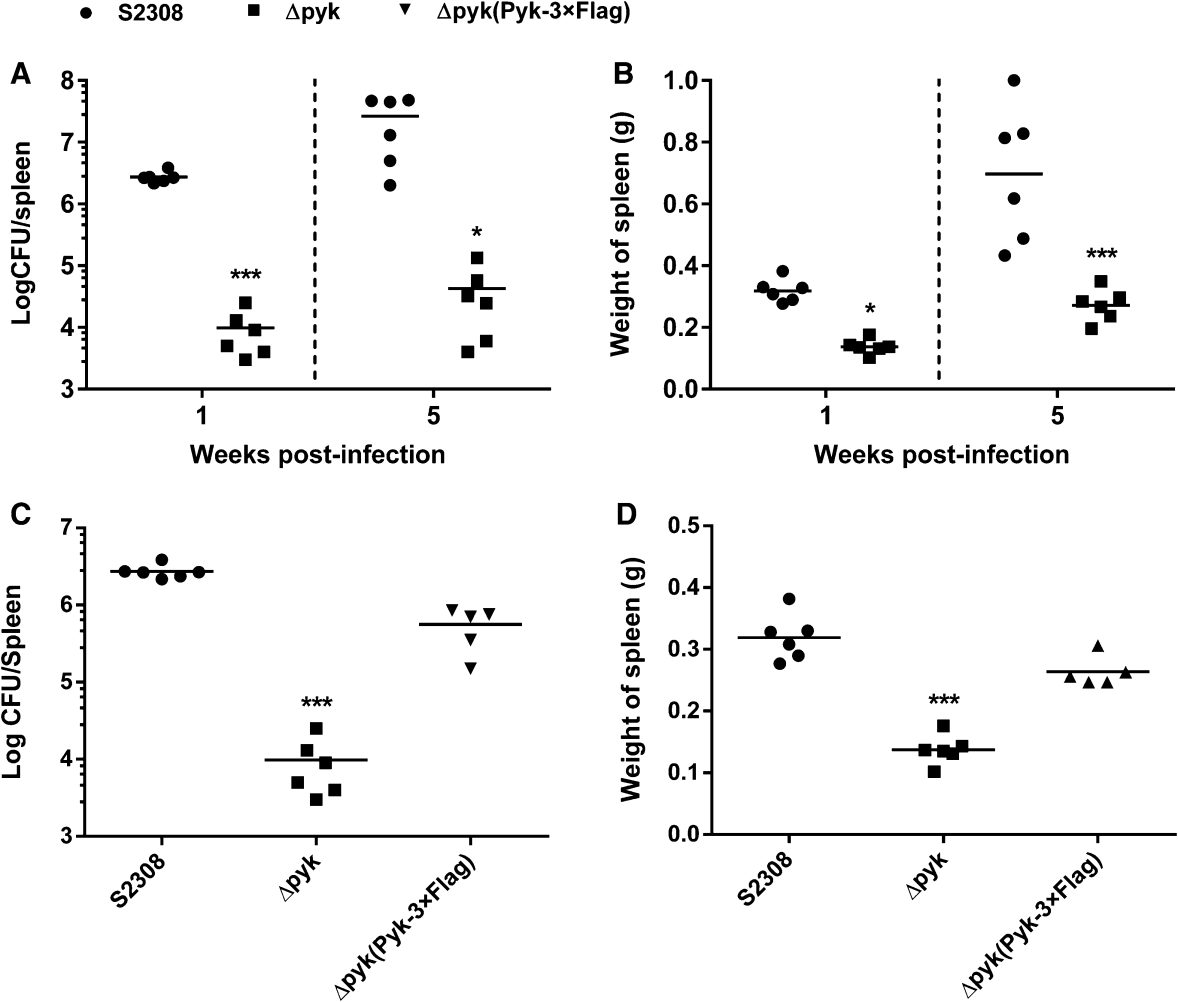
**Pyk is crucial for the virulence of** ***B. abortus*** **in mouse model of infection. A** Bacterial loads of the S2308 strain, the *pyk* mutant in the spleens of BALB/c mouse at 1 and 5 weeks post-infection. **B** Spleen weight of the S2308 strain and the *pyk* mutant infected mice at 1 and 5 weeks post-infection. **C** Bacterial loads of the S2308 strain, the *pyk* mutant and the complementation strain in the spleens of BALB/c mouse at 1 week post-infection. **D** Spleen weight of the S2308 strain, the *pyk* mutant and the complementation strain infected mice at 1 week post-infection *p ≤ 0.05; ***p ≤ 0.001.

The main manifestation of *Brucella* virulence is the ability to establish chronic infection in the host, therefore, we investigated the capacity of the *pyk* mutant to establish chronic infection in BALB/c mice. As shown in Figure 6A, the mutant exhibited significantly reduced level of spleenic colonization at 1 and 5 week post-infection. Similarly, the splenomegaly in mice infected with the mutant was less severe than that infected with the S2308 strain (Figure 6B). Besides, when harboring the pBBR1-*pyk*-3 × Flag plasmid, the *pyk* complementation strain restored the ability of splenic colonization and induced splenomegaly in infected BALB/c mice at 1 week post-infection (Figures 6C and D). These data suggested that the Pyk plays an important role in *Brucella* virulence.

## Discussion

Pyk in the glycolysis pathway converts PEP to pyruvate and releases one molecule of ATP. In minimal medium, the *pyk* mutant was not able to reach a similar level at stationary phase like the S2308 strain, and showed a marked growth defect (Figure 4B). However, in TSB, the mutant exhibited a slight growth defect at the exponential phase, but finally achieved a similar level at the stationary phase (Figure 4A). The growth of the *pyk* mutant was restored when pyruvate was added, which suggested that the loss of the metabolic product pyruvate is the main reason for defective *Brucella* growth. It was suggested that Pyk plays an important role in *Brucella* growth, especially under the condition of nutrition deprivation. In *Brucella*, it has been reported that gluconeogenesis pathway is very important for full virulence of *Brucella*, and the classical genes involved in this pathway, *ppdk* and *mae*, are responsible for the markedly reduced multiplication of the mutant within macrophages and virulence in mouse model [14]. These observations confirmed the deduction that amino acids could be the preferred carbon source in vivo, which requires a gluconeogenic mechanism [21]. However, some studies suggested the availability of sugars in the replicative niche of intracellular *Brucella*. Xavier et al. observed that glucose uptake is crucial for increased replication of *B. abortus* in alternatively activated macrophages and for chronic infection in a mouse model [22]. These results indicated that the glycolytic pathway may also play an important role in metabolism and virulence of intracellular *Brucella*. Pyk, as an important enzyme for the pyruvate-TCA cycle node, catalyzes PEP to pyruvate irreversibly as the last step of the glycolytic pathway, which plays a significant role in *Brucella* virulence. Our results are consistent with those reported for other pathogens, such as the enteric pathogen *Yersinia pseudotuberculosis*, as Pyk resulted in significantly reduced virulence of a *Yersinia* mutant in a mouse infection model [23].

In order to further explain the attenuated mechanism of the *pyk* mutant, we analyzed other biological properties of the mutant and found that the *pyk* mutant was more sensitive to H_2_O_2_ than the S2308 strain. In a further study, we used real-time qRT-PCR to analyze transcription levels of genes associated with antioxidant mechanisms, including *katA*, *sodC* and *oxyR*, but found no significant difference in expression levels between the *pyk* mutant and the S2308 strain (data not shown), suggesting that reduced resistance to oxidative stress of the *pyk* mutant was barely attributable to the conventional antioxidant mechanism. The change in the outer membrane was also shown to affect sensitivity to oxidative stress [24–26], so the liposaccharide integrity of the mutant was confirmed by silver-staining, but no difference was found between the mutant and the S2308 strain (data not shown). The role of Pyk in *B. abortus* to resist oxidative stress warrants further investigation. Another manifestation of *Brucella* virulence is the ability of intracellular survival within target cells. Once taken up, *Brucella* resides in a vacuole, designated as BCV. BCVs initially fuse with endosome, and then fuse with lysosome to some extent, acquire the lysosome marker LAMP-1. Meanwhile, the BCVs are acidified by lysosome, which is a key step the *Brucella* needs to express the important virulence components such as Type IV secretion system, to help it to escape the fusion of BCV and lysosome, and then the BCVs re-localize to endoplasmic reticulum, forming permissive replicative *Brucella* vacuole. Co-localization of BCV and LAMP-1 is a key marker for determining the efficiency of BCV excluding lysosome [27]. At late stage of *Brucella* trafficking within host cell, the *pyk* mutant barely excluded the lysosomal marker LAMP-1 (Figure 3), which explained the reason why the mutant was not able to replicate efficiently within macrophages. Therefore, we speculated the mechanism of virulence attenuation for the *pyk* mutant as defected growth ability under a condition of nutrition deprivation, reduced resistance to oxidative response, and enhanced fusion efficiency with lysosome within host cells.

In summary, this study illuminated that the carbon metabolism gene *pyk* plays important roles on *B. abortus* growth, especially under the condition of nutrition deprivation, intracellular survival, and establishment of chronic infection in mice. This study provides further insight into the role of Pyk on *B. abortus* virulence.
